# Occipital Seizures and Persistent Homonymous Hemianopia (HH) With MRI Subcortical T2 Hypointensity in a Newly Diagnosed Diabetic Patient

**DOI:** 10.7759/cureus.25648

**Published:** 2022-06-04

**Authors:** Duygu Engez, Nihan Hanife Yılmaz, Esma Nur Ekmekçi, İrem Sert, Guray Koc

**Affiliations:** 1 Department of Neurology, Ankara City Hospital, Ankara, TUR

**Keywords:** subcortical t2 hypointensity, electroencephalograph, homonymous hemianopia, hyperglycemia, occipital seizures

## Abstract

Non-ketotic hyperglycemia (NKH) can often cause seizures. Although these are usually in the form of focal seizures, occipital seizures have also been reported in case reports. Patients may present with complaints ranging from blurred vision and bright lights to homonymous hemianopia (HH) in occipital seizures due to hyperglycemia. Seizures can often be brought under control in a short time with good glycemic control. Seizures associated with NKH may cause subcortical T2 hypointensity on MRI in the occipital lobes and occipital epileptiform discharges on the electroencephalogram.

In this case study, we aim to present a newly diagnosed diabetes mellitus patient who had homonymous hemianopsia in his neurological examination, had imaging and electrophysiological findings consistent with his examination and clinical findings, was admitted 15 days after his symptoms started, and whose seizures could not be controlled by glucose regulation. In this context, we evaluated the literature and compared our case to other patients who required anti-seizure drugs, with the goal of emphasizing the need of early treatment in seizures caused by NKH.

## Introduction

Hyperglycemic seizures are considered one of the most severe neurological complications of diabetes mellitus [1]. Non-ketotic hyperglycemia (NKH) is a clinical syndrome of severe hyperglycemia without ketoacidosis. Patients with NKH have been shown to suffer seizures in 15-40% of cases [2]. While motor seizures were widespread, only a few cases of occipital seizures with or without hemianopia were reported [3]. T2-weighted and fluid-attenuated inversion recovery (FLAIR) subcortical hypointensity have been observed in association with NKH, and these findings are occasionally reversible [4]. The major treatment includes managing of hyperglycemia and ensuring enough hydration. The role of anti-seizure medications, on the other hand, is controversial [1].

We intend to present a case with occipital seizures and persistent homonymous hemianopia (HH) with MRI subcortical T2 hypointensity in a undiagnosed diabetic patient, as well as to assess other instances in the literature based on the findings of this patient.

## Case presentation

 A 56-year-old right-handed male with no known history of neurological diseases presented with the complaint of seeing flashing multicolored lights and geometric patterns akin to circles in his right visual field for 15 days. He also described a visual deficit in the right visual field. His previous medical history included hypertension, myocardial infarction, and a cardiac stent, and he was treated with acetylsalicylic acid 100 mg/day, prasugrel 10 mg/day, ramipril 5 mg/day, and isosorbide 20 mg/day. There was no previous history of seizures or migraines.

Seizures began with positive visual phenomena, such as bursts of lightning and colorful elements, followed by a red visual field. He saw simple objects in his right visual field most of the time, but he sometimes saw complicated images like a walking dog. Then seizures continued with the right deviation of eyes without any other motor component. Seizures lasted approximately two-three minutes and occurred in clusters. During the seizures, the patient was aware. The factors that triggered his seizure were investigated, including his infection status, psychological stress, extreme fatigue, and so forth. There was no indication of a relevant factor. His neurological examination revealed right homonymous hemianopsia.

**Figure 1:**
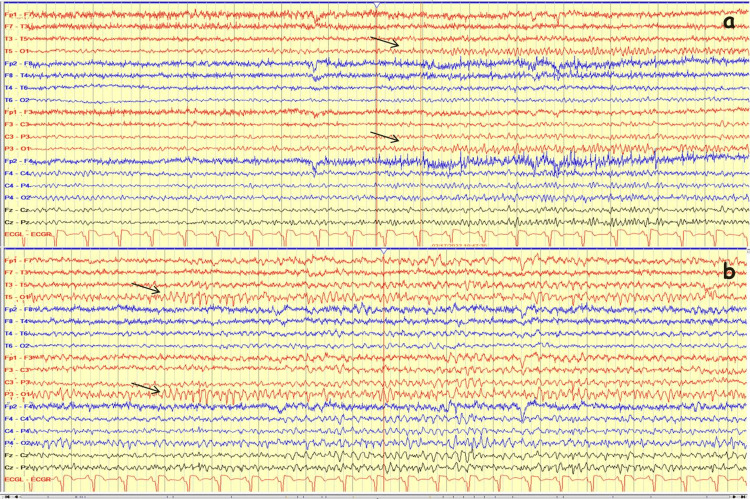
EEG on admission day A) Ictal EEG started with left occipital rhythmic alpha activity (arrow); B) the frequency of this activity reduced and the amplitude increased (arrow). Low frequency filter (LFF): 1 Hz; High frequency filter (HFF): 70 Hz; Sensivity: 7 µvolt/mm

**Figure 2:**
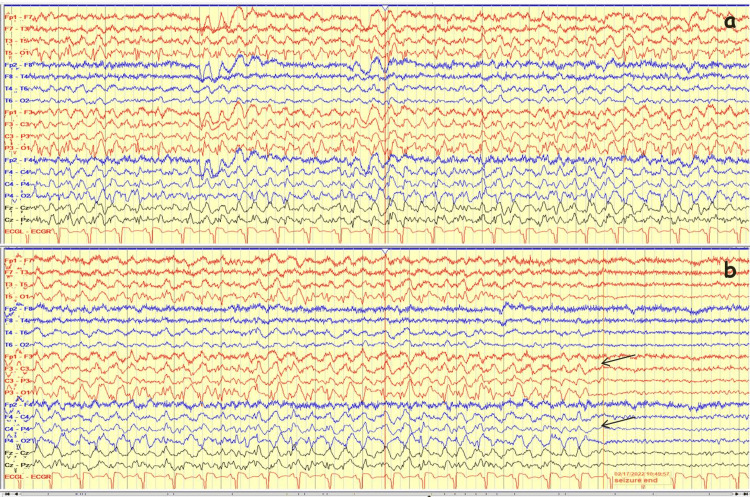
EEG on admission day (continued) A) Ictal activity spread to ipsilateral and contralateral regions and continued as rhythmic sharp and slow-wave;  B) All of the evolution criteria were completed. The ictal activity ended abruptly and postictal generalized attenuation was seen (arrow). The total seizure duration was 160 seconds. There were two additional ictal EEG recordings on seven hospital days with the same ictal EEG activity, and their durations were 150 and 165 seconds, respectively. There was no interictal epileptiform activity. Low frequency filter (LFF): 1 Hz; High frequency filter (HFF): 70 Hz; Sensivity: 7 µvolt/mm

**Figure 3:**
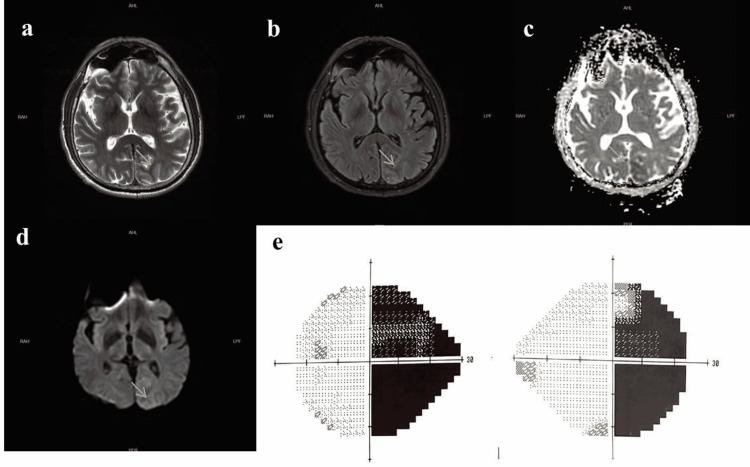
Patient's MRI and visual field test MRI showed a non-space-occupying T2 (a) and fluid-attenuated inversion recovery image (FLAIR) (b) sequences hypointense lesion of the subcortical white matter in the left occipital lobe (arrows). Apparent diffusion coefficient (ADC) signal (c) significantly decreased (arrow), diffusion-weighted imaging (DWI) signal (d) showed subtle gray matter hyperintense and white matter hypointense lesion (arrow). The visual field test was consistent with the patient’s right homonymous hemianopia (HH) (e).

First laboratory results of the patient included a blood glucose level of 337 mg/dL, a serum osmolarity level of 296.15 mosmol/L. The serum ammonia in the blood was level of 39.1 µmol/L. The urine ketone test was negative. Ketoacidosis did not exist, and the pH level was 7.418. The serum HbA1C level was 12.7%. Other electrolytes (Na: 133 mEq/L, K: 4.2 mEq/L, urea: 32 mg/dL, Cl: 100 mEq/L, Creatinine: 1.03 mg/dL, HCO_3_: 24.5 mmol/L) were normal. The occipital seizure was recorded on EEG with beginning ictal alpha activity on the left occipital region then frequency reduced to theta and delta, respectively. The amplitude increased and the ictal activity spread (Figures 1,2). The semiology of seizures was a deviation of the eye to the right without unawareness. Brain MRI revealed T2 and FLAIR hypointensities in the left occipital parasagittal subcortical area without contrast enhancement. Right homonymous hemianopsia was confirmed by a visual field test (Figure 3). Brain PET study was found normal.

 

The patient was diagnosed with diabetes mellitus and treated with short- and long-acting insulin therapy to provide glycemic control and appropriate hydration was given. Seizures persisted despite the treatment and achievement of glycemic control. Due to frequent seizures occurred throughout the hospitalization, carbamazepine was started at a dose of 400 mg/day. However, the seizures persisted, and two seizures with the same ictal activity and semiology were observed on control video EEG monitoring. The carbamazepine dose was increased to 800 mg/day. Because of the persistent seizures, levetiracetam (1000 mg/day) was added to the treatment. The seizures were controlled with the addition of levetiracetam to the treatment, and the patient was discharged with appropriate glycemic control. One month later, at the follow-up appointment, the patient was seizure-free. Moreover, his visual field was completely recovered.

## Discussion

Seizures due to hyperglycemia are often observed as epilepsia partialis continua, a kind of motor seizure [5,6]. Aside from these seizures, occipital seizures have been identified in NKH with glucose levels ranging from 200 mg/dL to above 500 mg/dL [7]. Occipital seizures caused by hyperglycemia are limited to case reports in the literature. The onset of most occipital seizures due to hyperglycemia is acute in the reported cases, and the use of anti-seizure drugs for seizure control was mostly unnecessary with the prompt glucose regulation performed in the acute period [2,8]. However, there were several cases in the literature that required anti-seizure drugs [2,3,5,7-14] (Table 1).

**Table 1:** Case reports of hyperglicemia-related occipital seizures and/or HH Abn.: abnormality; HH: homonymous hemianopia; GTC: generalized tonic clonic; E: ictal EEG activity; S: focal slow waves; e: interictal epileptiform discharges; A: EEG asymmetry; GC: glycemic control;  N/A: not available; T2WI/FLAIR  abn.: focal subcortical hypointensity on T2 weighted image and/or fluid-attenuated inversion recovery image; O: occipital ^a^ newly diagnosed diabetes mellitus; ^b^ Right temporo-occipital cortical thickening with T2 hyperintensity and subtle gadolinium enhancement of the right hippocampus

Age / Gender	Presentations	EEG	Glucose (mg/dL)	HbA1C (%)	Serum osmolarity (mOsm/kg)	Treatment	Time to treatment	T2WI/FLAIR Abn.	Reference
54/M^a^	Seeing round, colored flickering lights with right HH	S	645	14.4	297	GC	One week	+ (Left O)	Xiang et al [2]
50/F^a^	Diplopia, right HH, looking right side	E (Left O)	250	10.5	333	Carbamazepine, GC	About two days	-	Del Felice et al [3]
66/M	Right HH, right head turn and right beating nystagmus	E (Left O)	359	13.4	N/A	Phenytoin, lorazepam, GC	One week	+ (Left O)	Putta et al[5]
30/M^a^	Green-colored flashing lights in the left-sided visual field, left HH, left gaze, head deviation, GTC	E (Right O)	372	13.8	304	Phenytoin, midazolam, GC	One week	+ (Right O)	Hung et al [7]
52/F^a^	Episodic visual hallucinations	e (Right O)	310	14.4	295	Phenytoin, GC	Two weeks	+ (Right O)	Hung et al [7]
83/M	Flashes of green and blue lights, HH, arm jerks on the left side	E (Left O)	639	N/A	316	GC	About two days	N/A	Moien-Afshari et al [8]
53/M^a^	Visual floaters and left HH, left side ptosis, subtle left upper extremity pronator drift	Normal	630	14.2	322	GC	Same day	-	Kashani et al [9]
61/F^a^	Left HH	S (Right hemisphere)	943	N/A	341	GC	About five days	+ (Right O)	Guez et al [10]
53/M	Right HH, GTC seizure	E (Left O)	581	11.4	N/A	Phenytoin, oxcarbazepine, GC	N/A	+ (Left parieto-occipital)	Nissa et al [11]
65/M^a^	Lower right-side pastel-colored flashing lights, quadrantanopsia	A (Left O)	370	11.4	326	GC	About two weeks	+ (Left O)	Sasaki et al [12]
45/M	Blurred vision, behavioral arrest left HH, left eye and head deviation	E (Right O)	267	N/A	293	Valproic acid, levetiracetam, oxcarbazepine, GC	N/A	^b ^Right temporo-occipital	Stayman et al [13]
60/M^a^	Left side colored spots and HH, left end gaze nystagmus	E (Right temporo -occipital)	320	N/A	290	Levetiracetam, oxcarbazepin GC	N/A	+ (Right O)	Stayman et al [13]
69/M^a^	Visual disturbances described as “green circles” in the right lower binocular field, right HH	E (Left O)	487	N/A	315	Phenytoin, GC	Five days	-	Stayman et al [13]
62/F	Colour lights and flashes, left inferior homonymous quadrantanopia	N/A	623	13.3	N/A	GC	Several days	-	Lopez-Amoros et al [14]
56/M^a^	Right side colorful flashes and geometric patterns complex visual hallucination, HH	E (Left O)	337	12.7	296	Carbamazepine, levetiracetam GC	Fifteen days	+ (Left O)	Our case

In our case, glycemic control didn't cease the seizures. Therefore, anti-seizure medication was administered. It is essential to treat this case as epilepsia partialis continua and suggest anti-seizure drugs as first-line treatment besides correcting hyperglycemia as a standard practice. In isolated non-recurrent metabolic seizures, correction of metabolic abnormality may be sufficient. However, in cases with prolonged metabolic abnormality and MRI changes anti-seizure drugs are essential. We believe that this situation could be caused by several reasons. First, our patient was admitted to the hospital several days after his complaints began. His symptoms had persisted for 15 days at the time of diagnosis and treatment. Secondly, our patient had a significantly elevated HbA1C (12.7 %) which has shown us that, he had an uncontrolled hyperglycemia and was not diagnosed with diabetes mellitus for a long period. In the literature, there are patients who presented with occipital seizure and were diagnosed with diabetes mellitus, like our case (Table 1). This arises the questions whether the acute hyperglycemia or the long exposure of hyperglycemia cause the occipital seizure. According to Hung et al., chronic exposure to uncontrolled hyperglycemia, as shown by raised HbA1c, is more crucial in the development of these seizures than acute NKH [7].

Abnormal glucose level may trigger seizures. Both in vivo and in vitro experimental studies indicated that a threshold glucose concentration is required to support synaptic transmission. High extracellular glucose, on the other hand, has been linked to neuronal hyperexcitability. All of this demonstrates the significance of glucose balance for normal nerve conduction. The mechanism of seizures due to hyperglycemia is still unknown [15]. Potential reasons for the etiology of seizures due to hyperglycemia have been evaluated as a decrease in the threshold of neuronal excitability due to high glucose levels, deterioration of the intracellular glutamate, and gamma aminobutyric acid (GABA) balance in favor of activation. Many studies have shown the physiological role of insulin in decreasing the excitability of neural networks [16]. In a recent study, low-dose intranasal insulin significantly reduced the duration and frequency of provoked seizures in mouse models [17].

In addition to inhibitory and excitatory mechanisms, it has been suggested that potassium-dependent adenosine triphosphate (ATP) channels affect hyperglycemia-induced seizures [18,19]. The termination of the seizures after the anti-seizure drug with levetiracetam ingredient, which affects the potassium channels of the patient, also strengthened the idea that K-ATP channels have a role in the mechanism of action on seizures due to hyperglycemia. At the presynaptic level, levetiracetam not only directly binds to the synaptic vesicle protein SV2A but also paradoxically prevents repeated constant stimuli by lowering voltage-dependent potassium currents. We think that controlling our patient's seizures after levetiracetam, which is also effective on potassium channels, supports the mechanism of action of K-ATP channels in seizures due to hyperglycemia. In our treatment, we did not consider choosing phenytoin, as it could have aggravated hyperglycemia-related seizures by blocking insulin secretion and creating insulin resistance [20].

Hyperglycemia may have triggered epileptogenesis by causing neuronal damage and may have led to resistant seizures by complicating seizure control. Paoletti et al. presented a case of right temporal lobe seizure caused by hyperglycemia, whose seizures lasted for several weeks and required anti-seizure drug therapy [4]. Nissa et al. also presented a case of persistent HH lasting almost 3 days, which improved after the addition of a second anti-seizure drug (phenytoin followed by oxcarbazepine) with glycemic control. They noted that this prolonged visual deficit had been previously reported in the postictal Todd phenomenon in hyperglycemia or in epilepsy partialis continua [11].

It has been shown that elevations in blood glucose levels may cause T2 subcortical hypointensities in MRI. The most plausible reason for this subcortical occurrence, is a brief increase and/or buildup of free radicals that may affect T2 signal [4]. Other possible reasons are iron deposition caused by hypoxic-ischemic injury, transient seizure effect, and intracellular dehydration [11]. The resistant right homonymous hemianopsia of our patient was supported by the T2 and FLAIR hypointensities in the left occipital parasagittal subcortical area. This lends credence to the idea of Sasaki et al. that iron buildup caused by excitotoxic damage from early cortical ischemia or seizures is a mechanism for subcortical T2 hypointensity [12].

## Conclusions

Occipital seizures and persistent hemianopia might appear as the initial symptom of NKH, as in our case. If treatment begins immediately after the seizures, glycemic control may be sufficient to resolve the seizures. However, if seizures continue for an extended period, anti-seizure medication treatment may be required. In these cases, anti-seizure drugs that impact K channels may be a feasible alternative. Prompt diagnosis and treatment are critical in these situations to avoid persistent seizures and visual field abnormalities.
